# Best Practices for Long-Term Monitoring and Follow-Up of Alemtuzumab-Treated MS Patients in Real-World Clinical Settings

**DOI:** 10.3389/fneur.2019.00253

**Published:** 2019-03-22

**Authors:** Krista Barclay, Robert Carruthers, Anthony Traboulsee, Ann D. Bass, Christopher LaGanke, Antonio Bertolotto, Aaron Boster, Elisabeth G. Celius, Jérôme de Seze, Dionisio Dela Cruz, Mario Habek, Jong-Mi Lee, Volker Limmroth, Sven G. Meuth, Celia Oreja-Guevara, Patricia Pagnotta, Cindy Vos, Tjalf Ziemssen, Darren P. Baker, Bart Van Wijmeersch

**Affiliations:** ^1^Vancouver Coastal Health, Vancouver, BC, Canada; ^2^University of British Columbia, Vancouver, BC, Canada; ^3^Neurology Center of San Antonio, San Antonio, TX, United States; ^4^North Central Neurology Associates, Cullman, AL, United States; ^5^Azienda Ospedaliero Universitaria San Luigi, Orbassano, Italy; ^6^OhioHealth Neurological Physicians, Columbus, OH, United States; ^7^Oslo University Hospital Ullevål and Institute of Clinical Medicine, University of Oslo, Oslo, Norway; ^8^Clinical Research Center (CIC), INSERM 1434, Strasbourg University, Strasbourg, France; ^9^Imperial College Healthcare NHS Trust, London, United Kingdom; ^10^University of Zagreb, School of Medicine and University Medical Center, Zagreb, Croatia; ^11^Stanford Healthcare, Palo Alto, CA, United States; ^12^Klinik für Neurologie und Palliativmedizin, Cologne, Germany; ^13^Clinic of Neurology with Institute of Translational Neurology, University Hospital Müenster, Müenster, Germany; ^14^El Instituto de Investigación Sanitaria del Hospital Clínico San Carlos, Hospital Clínico San Carlos, Madrid, Spain; ^15^Neurology Associates, Maitland, FL, United States; ^16^Revalidatie & MS Centrum, Overpelt, Belgium; ^17^Center of Clinical Neuroscience, University Clinic Carl Gustav Carus, Dresden, Germany; ^18^Sanofi, Cambridge, MA, United States; ^19^Rehabilitation & MS-Centre Overpelt, Hasselt University, Hasselt, Belgium

**Keywords:** relapsing-remitting multiple sclerosis, disease-modifying therapy, alemtuzumab, anti-CD52 monoclonal antibody, monitoring, autoimmune events, best practices, real-world settings

## Abstract

Multiple sclerosis (MS) is a chronic autoimmune neurological disease that typically affects young adults, causing irreversible physical disability and cognitive impairment. Alemtuzumab, administered intravenously as 2 initial courses of 12 mg/day (5 consecutive days at baseline, and 3 consecutive days 12 months later), resulted in significantly greater improvements in clinical and MRI outcomes vs. subcutaneous interferon beta-1a over 2 years in patients with active relapsing-remitting MS (RRMS) who were either treatment-naive (CARE-MS I; NCT00530348) or had an inadequate response to prior therapy (CARE-MS II; NCT00548405). Efficacy with alemtuzumab was maintained over 7 years in subsequent extension studies (NCT00930553; NCT02255656), in the absence of continuous treatment and with a consistent safety profile. There is an increased incidence of autoimmune events in patients treated with alemtuzumab (mainly thyroid events, but also immune thrombocytopenia and nephropathy), which imparts a need for mandatory safety monitoring for 4 years following the last treatment. The risk management strategy for alemtuzumab-treated patients includes laboratory monitoring and a comprehensive patient education and support program that enables early detection and effective management of autoimmune events, yielding optimal outcomes for MS patients. Here we provide an overview of tools and techniques that have been implemented in real-world clinical settings to reduce the burden of monitoring for both patients and healthcare providers, including customized educational materials, the use of social media, and interactive online databases for managing healthcare data. Many practices are also enhancing patient outreach efforts through coordination with specialized nursing services and ancillary caregivers. The best practice recommendations for safety monitoring described in this article, based on experiences in real-world clinical settings, may enable early detection and management of autoimmune events, and help with implementation of monitoring requirements while maximizing the benefits of alemtuzumab treatment for MS patients.

## Introduction

Multiple sclerosis (MS) is a chronic autoimmune neurological disease that is the leading cause of disability in young adults (1). It is frequently characterized by relapses and remissions with variable sequelae, which may persist and contribute to progressive and irreversible long-term disability (2). The underlying pathology of MS is typically marked by acute lymphocytic inflammation that leads to neuronal demyelination, neurodegeneration, axonal damage, and to the eventual formation of lesions within the central nervous system (3, 4). Alemtuzumab is a humanized anti-CD52 monoclonal antibody approved for the treatment of patients with active relapsing-remitting MS (RRMS) defined by clinical or imaging features. It is administered as intravenous infusions of 2 courses of 12 mg/day on 5 consecutive days at baseline, and on 3 consecutive days 12 months later (5, 6). In Australia and the European Union, 2 additional courses may be given as needed for clinical and MRI activity (3 consecutive days, ≥12 months after the prior course) (6, 7). The efficacy and safety of alemtuzumab were established in 3 rater-blinded, comparative clinical trials in patients who were either treatment-naive (phase 2 CAMMS223; NCT00050778; and phase 3 Comparison of Alemtuzumab and Rebif Efficacy in Multiple Sclerosis [CARE-MS] I; NCT00530348) (8, 9) or had an inadequate response to prior therapy (phase 3 CARE-MS II; NCT00548405) (10).

Alemtuzumab significantly reduced relapses compared with subcutaneous interferon beta-1a (*p* < 0.001 in all 3 clinical trials) and improved disability over 2 years (*p* < 0.001, and *p* = 0.008 in the CAMMS223, and CARE-MS II trials, respectively). In extension studies (NCT00930553, NCT02255656 [TOPAZ]), the efficacy of alemtuzumab was maintained over at least 7 years, with many patients (61% from CARE-MS I and 52% from CARE-MS II) receiving no additional courses of alemtuzumab (11–15). Although the exact mechanism of action has not been fully elucidated, alemtuzumab efficacy may be due to the selective depletion and distinct pattern of repopulation of circulating CD52-expressing T and B lymphocytes (16, 17). During repopulation, there is a relative increase in regulatory T cells which results in a shift from pro- to anti-inflammatory cytokine profiles driven by differential reconstitution of T-cell subsets, potentially leading to a rebalancing of the immune system (18–20). Increased secretion of neurotrophic factors by the regenerating immune cells may have a neuroprotective effect, and provide an explanation, at least in part, for the sustained disability improvements in alemtuzumab-treated patients (21).

The most common adverse events (AEs) associated with alemtuzumab are infusion-associated reactions and infections, with the highest rates occurring after the first treatment course and declining thereafter (13). Alemtuzumab is also associated with an increased risk for autoimmune events, including thyroid disease, immune thrombocytopenia (ITP), and nephropathies, which can manifest months to years after treatment. Table 1 shows the incidence of these events in the clinical development program and in postmarketing surveillance, suggesting that the incidence is not higher in real-world settings. Because of the potential for these autoimmune events, alemtuzumab is only available through the Risk Evaluation and Mitigation Strategy program in the United States and Canada, and the Risk Management Program in Europe (25, 26). These programs include monthly urinalysis and blood testing for renal and hematological monitoring, and quarterly thyroid function monitoring for 48 months after the last alemtuzumab infusion, as well as physician and patient education about signs and symptoms of these autoimmune events.

**Table 1:** Clinical trial incidence, post-marketing frequency, and signs and symptoms of key autoimmune events after treatment with alemtuzumab.

**Key autoimmune AE^a^**	**Incidence in clinical development program (alemtuzumab 12-mg treatment arm) (13)**	**Estimated frequency in post-marketing settings^b^ (22)**	**Signs and symptoms**
Thyroid events	42^c^	Not available	Overactive thyroid: diaphoresis, unexplained weight loss, eye swelling, nervousness, tachycardia Underactive thyroid: unexplained weight gain, feeling cold, increased fatigue, newly occurring constipation
Immune thrombocytopenia	2.0^d^	0.72	Bruising, petechiae, purpura, mucosal bleeding, increased menses, hematuria, melena
Nephropathy	0.27	0.17	Blood in the urine (urine may be red or tea-colored), swelling in legs and feet, hemoptysis

a *Does not include other rare autoimmune AEs (e.g., alopecia, acquired hemophilia A) that have been reported in alemtuzumab-treated patients (23, 24)*.

b *Calculated as number of cases/total number of patients (n = 18,561) treated through December 31, 2017*.

c *Over 6 years in the pooled CARE-MS patients who received alemtuzumab 12 mg (n = 811)*.

d *Over 5 years of total follow-up for CARE-MS studies and 8 years for CAMMS223 study in patients who received alemtuzumab 12 mg (n = 1,217)*.

*AE, adverse event; CARE-MS, Comparison of Alemtuzumab and Rebif Efficacy in Multiple Sclerosis*.

Monitoring can be challenging in clinical practice. As such, various monitoring tools and techniques have been developed in real-world clinical settings that may help to reduce the burden of monitoring and maximize the treatment benefit for patients receiving alemtuzumab. Although resources and strategies for patient management vary within individual practices in different countries, this article aims to provide an overview of these tools and techniques and offers perspectives from MS clinicians in various clinical settings on best practices to optimize patient care.

### Strategies and Tools to Assist With Monitoring Alemtuzumab-Treated Patients in the Clinic

#### Pre-treatment Preparation

##### Patient selection

A patient's commitment to adhere to the risk management program, including the level of cognitive function and ability to understand treatment benefits vs. risks, should be considered before initiating treatment with alemtuzumab. The decision to initiate alemtuzumab treatment should be made only after ensuring that patients understand the need for long-term monitoring, and/or if patients have family and community support to help comply with monitoring.

##### Patient education and lifestyle management

The benefits of patient education and engagement in the management of MS are well documented (27–30). National Institute for Clinical Excellence guidelines and the European MS Platform's Code of Good Practice recommend implementing an education program that incorporates information about the disease, and provides guidance on the level of communication and emotional support while encouraging patient autonomy/self-management (31, 32). The MS Brain Health initiative, which emerged from an evidence-based international consensus report (33), recommends a “brain-healthy” lifestyle as part of a comprehensive approach to treatment and management of MS. Given that cardiovascular fitness correlates with brain volume and cognitive reserve (34), patients should be advised to incorporate aerobic exercise into their daily routines. They should also be encouraged to stop smoking to prevent excessive reduction in brain volume and worsening of disability progression (35–37), and to limit the use of alcohol to help maintain and maximize lifelong brain health.

**Figure 1:**
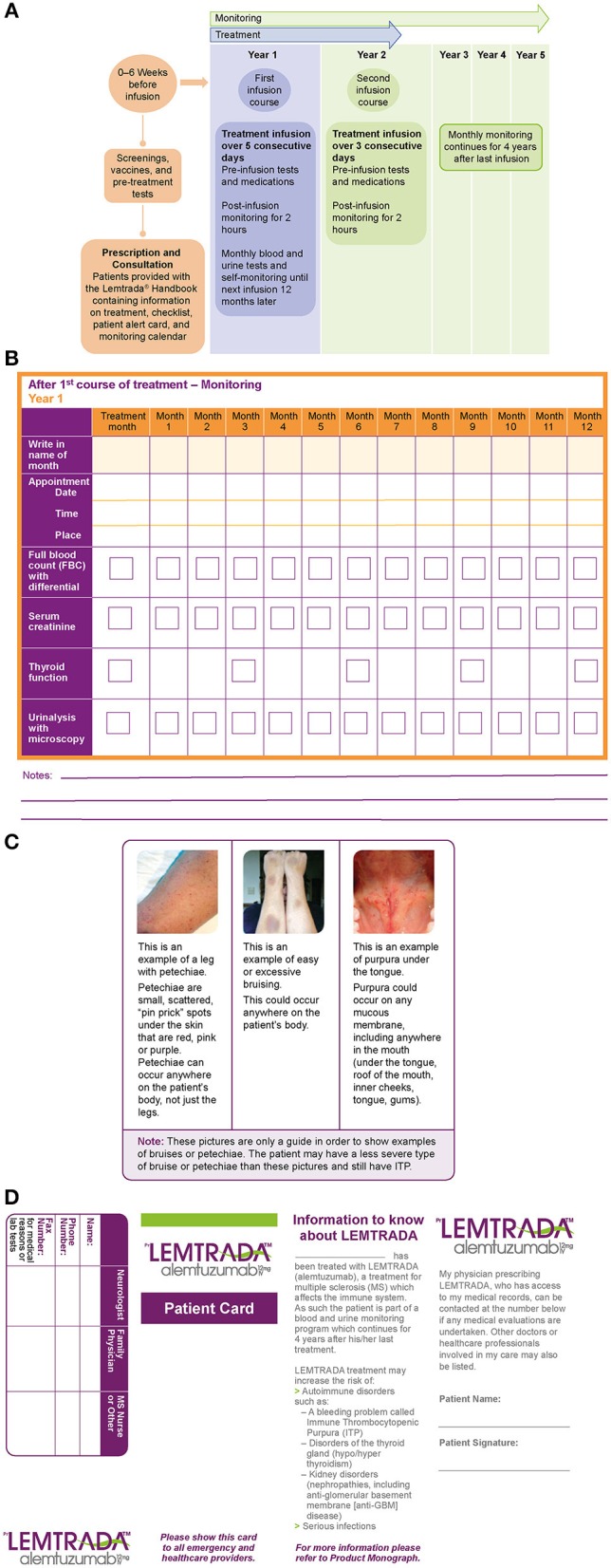
**(A)** Alemtuzumab dosing regimen and monitoring, **(B)** patient monitoring calendar, **(C)** patient reference guide with examples of immune thrombocytopenia (ITP) skin conditions, and **(D)** patient alert card.

As soon as the decision has been made to initiate treatment with alemtuzumab, patients receive various educational materials provided by the drug manufacturer, including a patient handbook that contains information on support services, treatment, and monitoring (Figures 1A,B). Patients are educated about the signs and symptoms of potential thyroid disorders, ITP, and nephropathies, and are given images of relevant AEs for visual reference (e.g., Figure 1C). Patients are also provided with an alert card containing helpful information for their regular and emergency healthcare providers (HCPs) (Figure 1D), which is particularly important if after-hours care is not available in the prescribing physician's office. In addition to educational materials provided by the drug manufacturer, clinical practices may consider developing their own information packages, as appropriate, including the use of social media (e.g., YouTube), highlighting the treatment benefits and potential side effects. However, as the veracity of online material is variable, scrutiny before recommending various platforms is pertinent.

It is crucial for HCPs to discuss the importance of monitoring with patients, as some patients may not understand the need to continue monitoring for an extended period after the completion of the active treatment phase. HCPs should emphasize that monitoring is for the benefit of the patient and that it can help with early detection and management of potential side effects, thereby ensuring that the positive effects of treatment are not circumvented. Additionally, HCPs may remind patients that many of the other MS therapies require daily to monthly administration, which is avoided with alemtuzumab. Some clinical practices request patients to sign an informed consent form, documenting that they have received education about the benefits and risks of treatment and are aware of their responsibilities for monitoring and follow-up, whereas other practices (in the UK and Belgium, for example) mail the alemtuzumab education materials directly to the patient's general practitioner (GP) to help with post-treatment monitoring. Lastly, patients should be advised to schedule their blood and urine tests at locations that are most convenient to them, including outpatient clinics, hospitals, or GPs office (if the latter provides these services). In some areas, clinical practices can also arrange for home visits by nursing staff to carry out monthly blood and urine tests.

#### Post-treatment Follow-Up

##### Patient support tools for monitoring

The alemtuzumab patient support program, MS One-to-One, is available to MS patients and their HCPs in North America, Europe, and other countries (Table 2) (38). It offers access to a network of nurses to assist with MS education and information on alemtuzumab. It also includes a platform for patients to share their experiences on living with MS, and provides links to support groups and patient advocacy organizations such as the MS Association of America and the National MS Society (39, 40). The MS One-to-One program has been used to assist with monitoring compliance and follow-up, particularly in MS clinics with large numbers of alemtuzumab-treated patients and in neurologists' offices with limited in-house support staff.

**Table 2:** Patient support tools for monitoring and follow-up after treatment with alemtuzumab.

**Program**	**Country**	**Scope**	**Contact information**
MS One-to-One	United States Canada Various countries in Europe South Africa	Assists with tracking results and monitoring compliance	www.msonetoone.com http://mylemtrada.ca http://msonetoone.eu https://msonetoone.co.za/patient/login#/
MSIS-29	UK	A 29-item, patient-reported questionnaire that assesses the physical and psychological impact of MS	https://www.mstrust.org.uk/sites/default/files/MSIS-29.pdf
Smartphone applications Examples: • MSAA' s My MS Manager™ • CareZone • LEMTRADA® MyLink • LEMCHECK™	United States and various countries in Europe	• Track patients' monitoring appointments • Access online links to alemtuzumab monitoring calendar • Provide information on signs and symptoms of autoimmune AEs • Send e-mail/text reminders to: ➣Comply with monthly tests (e.g., blood and urine) ➣Self-check for signs and symptoms of AEs Share findings with HCPs	
Lemtracks (“Your Lemtrada” in Italy)	Various countries in Europe	Assists HCPs and MS nurses with appointment reminders and sends alerts for missed appointments and abnormal test results	
LemMon	Belgium	Obtains blood and urine samples from patients in their homes	http://www.remedus.be/nl

*AE, adverse event; HCP, healthcare provider; MSAA, Multiple Sclerosis Association of America; MSIS-29, Multiple Sclerosis Impact Scale-29*.

Many clinics have developed web-based portals that facilitate communication between the patient and his/her clinical support team (41). These portals may include patient questionnaires to self-report new symptoms and disease impact on their daily quality of life, which in turn may prompt HCP referrals to other healthcare services. In the UK and other European countries, clinicians use the Multiple Sclerosis Impact Scale-29, a 29-item self-report rating scale for measuring the physical and psychological impact of MS (Table 2) (42, 43). Results from this online questionnaire are transferred to a web portal that can be accessed by HCPs within a given geographic area. Asymptomatic patients who do not need face-to-face consultation are followed up within 3–6 months. The nursing staff also conducts “virtual” consultations via Skype or telephone, thus providing patients with convenient and flexible alternatives to office visits.

In several European countries, as well as in the United States, alemtuzumab-treated patients can use smartphone applications to set up reminders for complying with monthly tests, to identify the signs and symptoms of side effects through self-check, and to access MS-related health information (Table 2). HCPs have begun integrating e-mail and text messaging into their clinical practices, and are providing online links to tools such as alemtuzumab monitoring calendars to help patients track their appointments.

Lemtracks is a web-based, electronic patient reminder system available to MS centers across Europe that supports alemtuzumab monitoring requirements (Table 2). It allows HCPs and MS nurses to send appointment reminders to patients and, importantly, alerts them to missed appointments and abnormal lab results.

In Belgium, clinical practices request an independent nurse service (e.g., LemMon) to obtain blood and urine samples from patients in their homes (Table 2). During their home visits, nurses educate patients on the importance of routine monitoring and self-checks for signs and symptoms of AEs. They also report the occurrence of possible AEs to the MS clinic, and alert the clinic when patients miss appointments. In the UK, monitoring nurses are in charge of performing the monthly monitoring tests in hospitals. If the tests are done in the GP's clinic, the monitoring nurses coordinate with the clinic as needed to ensure timely receipt of laboratory results for evaluation and follow-up. Other clinical practices in North America and Europe collaborate with nurses and social workers for follow-up and laboratory test reminders.

Patient support tools may vary by institution, country, and local regulations. For example, in Norway, legal regulations require HCPs to access data only from the patients' hospital records, which may limit the use of different methods for follow-up. Clinical practices would thus benefit from having dedicated nursing staff adequately trained to care for patients with MS, assess patient symptoms, detect potential AEs and abnormal laboratory results, and ensure careful record-keeping.

##### Patient support tools for management of laboratory test data

**Figure 2:**
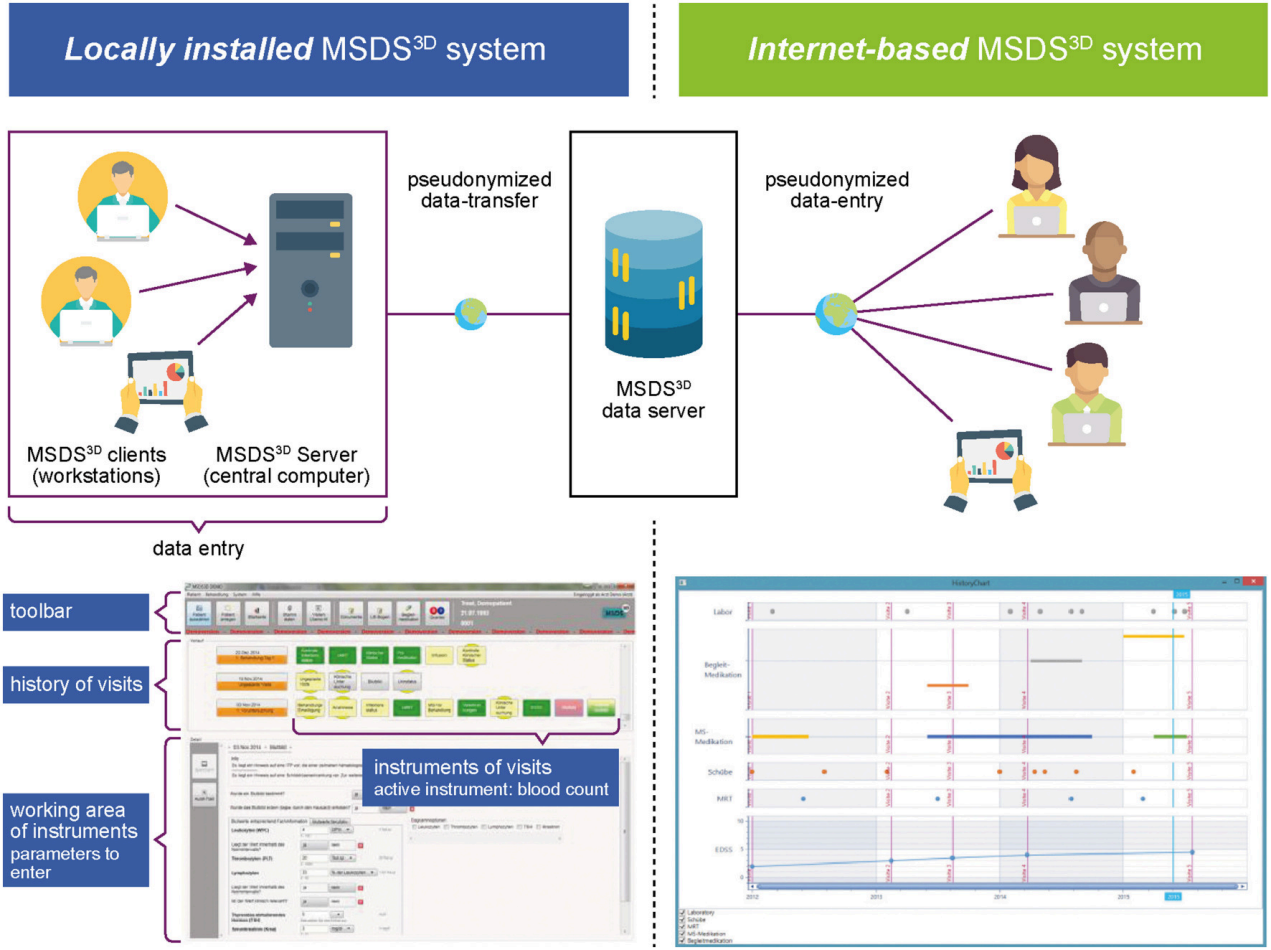
MSDS^3D^ patient data management system. Reprinted from Ziemssen et al. (49). Creative Commons Attribution 4.0 International Public License.

Several MS-specific networks and databases are available that can facilitate patient data management (44–47). The MS-Documentation System: 3-Dimensional—Physicians, Nurses, and Patients (MSDS^3D^) (48, 49) is a widely used electronic management system for MS patients in Germany that can integrate data generated by physicians, nurses, and patients (Figure 2). Laboratory data collected from different sites can be transmitted to a central service for automatic processing. The MSDS^3D^ platform also allows post-treatment monitoring of routine blood tests and immunologic samples, sending automatic e-mail notifications to HCPs. Patients' results are displayed in a progression graphic, providing a long-term overview of their condition. Other functionalities of the MSDS^3D^ system include clinical advice on difficult cases from experts who have restricted access to patient MRI data from external MRI reading centers, and collection of post-authorization safety data (50).

An MSDS^3D^ module for patients treated with alemtuzumab is currently being used in the TREAT-MS study (49). It is designed to connect all the HCPs responsible for the individual MS patient and enable comprehensive healthcare management by capturing patient data from the time of the last alemtuzumab treatment. Based on the patient data, MSDS^3D^ generates prospective checklists for physicians and patients before treatment initiation. These checklists would guide and facilitate the next steps in the treatment schedule, including generating health forms for insurance purposes. The structured interface of the MSDS^3D^ system can potentially support physicians and nurses to monitor alemtuzumab-treated patients effectively.

Many clinics across Australia use an automated clinical decision support system to implement the risk management program for MS patients treated with alemtuzumab (51). This computerized system is designed to track the pathology reports of alemtuzumab-treated patients and provide customizable alerts for abnormalities in identified risks.

In Canada, some clinical practices are using an automated, integrated, healthcare data management system developed by Excelleris™ Technologies (52) to monitor patients after treatment with alemtuzumab. Excelleris is a medical platform that standardizes health information from various sources and transmits laboratory data to clinicians. Building on this medical platform, clinicians have developed an automated alert program for ensuring compliance with monitoring monthly tests and screening for autoimmune events specific to alemtuzumab-treated patients. The platform is designed to harvest laboratory results, regardless of the laboratory location or the prescribing physician, and immediately trigger e-mail/text alerts for both clinicians and patients if a significant abnormality is identified within the program's parameters. This alemtuzumab automated alert platform contains other features that facilitate monitoring and follow-up, including a clinician portal that compiles patient information, graphs and analyzes test results and/or patterns over time, identifies the infusion dates, and allows communication between HCPs within the portal to respond to AEs.

**Figure 3:**
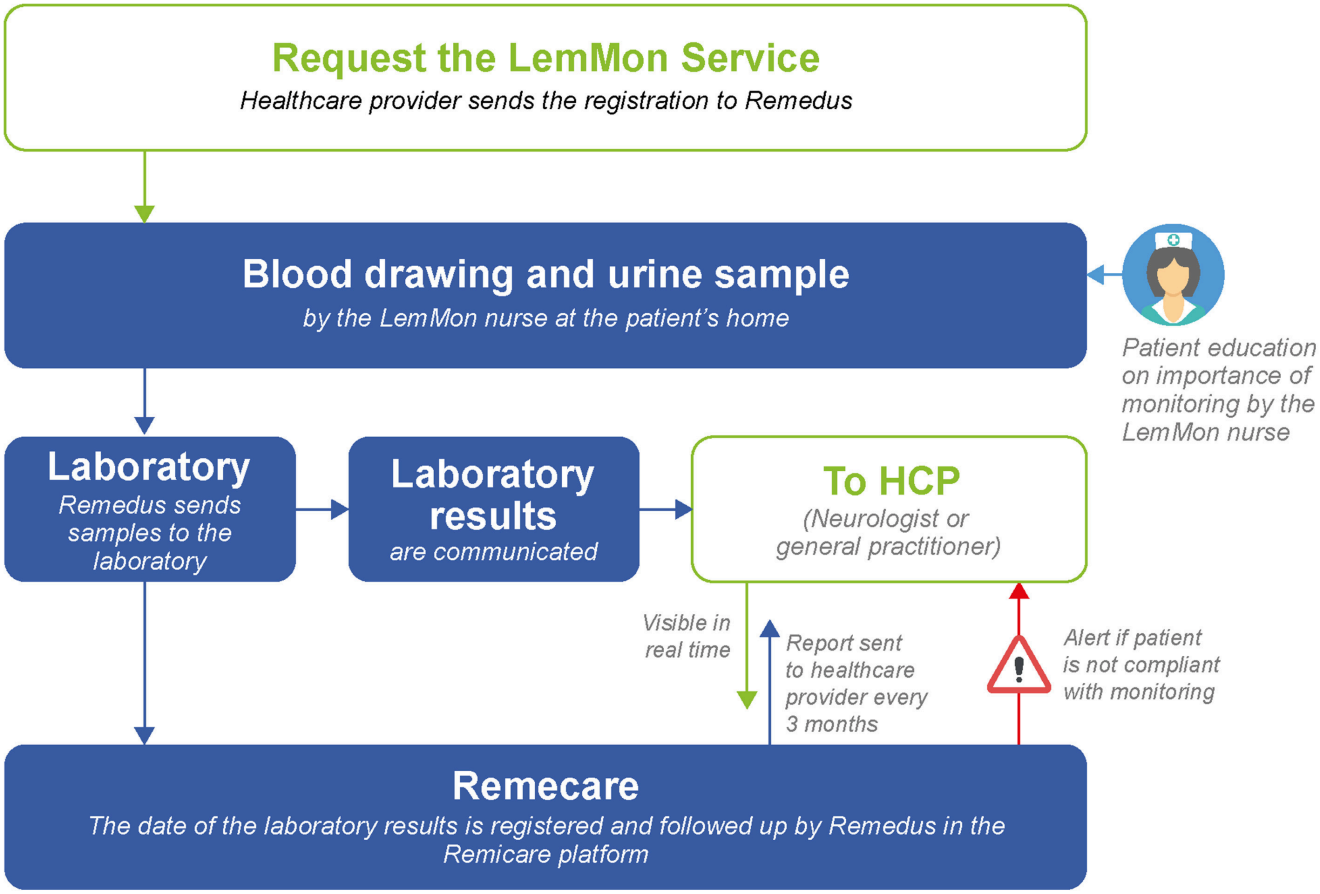
The Remedus data management platform.

Remedus is an online platform used in MS clinical practices across Belgium to communicate laboratory test results to HCPs (a neurologist or a GP) in real-time (Figure 3) (53). The database stores patient data along with the date of testing, and generates reports for HCPs every 3 months. Any abnormal result or delay in patient compliance triggers an alert for HCPs, allowing them to follow up with the patient.

### Addressing Patient Non-compliance

In real-world clinical settings, even with the implementation of best practices for monitoring, some patients may not be compliant. Alemtuzumab-treated patients who do not have any evidence of disease activity and have not received additional treatment since their initial 2 courses of alemtuzumab, may be at particular risk for non-compliance with monitoring. Additionally, patients may become worn out by the requirement for frequent blood draws. Cognitive deficits and loss of family support may also contribute to non-compliance.

If a patient becomes non-compliant, HCPs and the nursing staff should take steps to determine an acceptable solution to re-establish compliance. This may include in-home lab collection, personal follow-up, seeking assistance from patient's GP and family members/community, tailoring reminder systems to suit the patient's lifestyle, and reiterating the importance of monitoring, while highlighting the treatment benefits vs. risks.

### Practical Guidance for Management of Autoimmune Events

**Figure 4:**
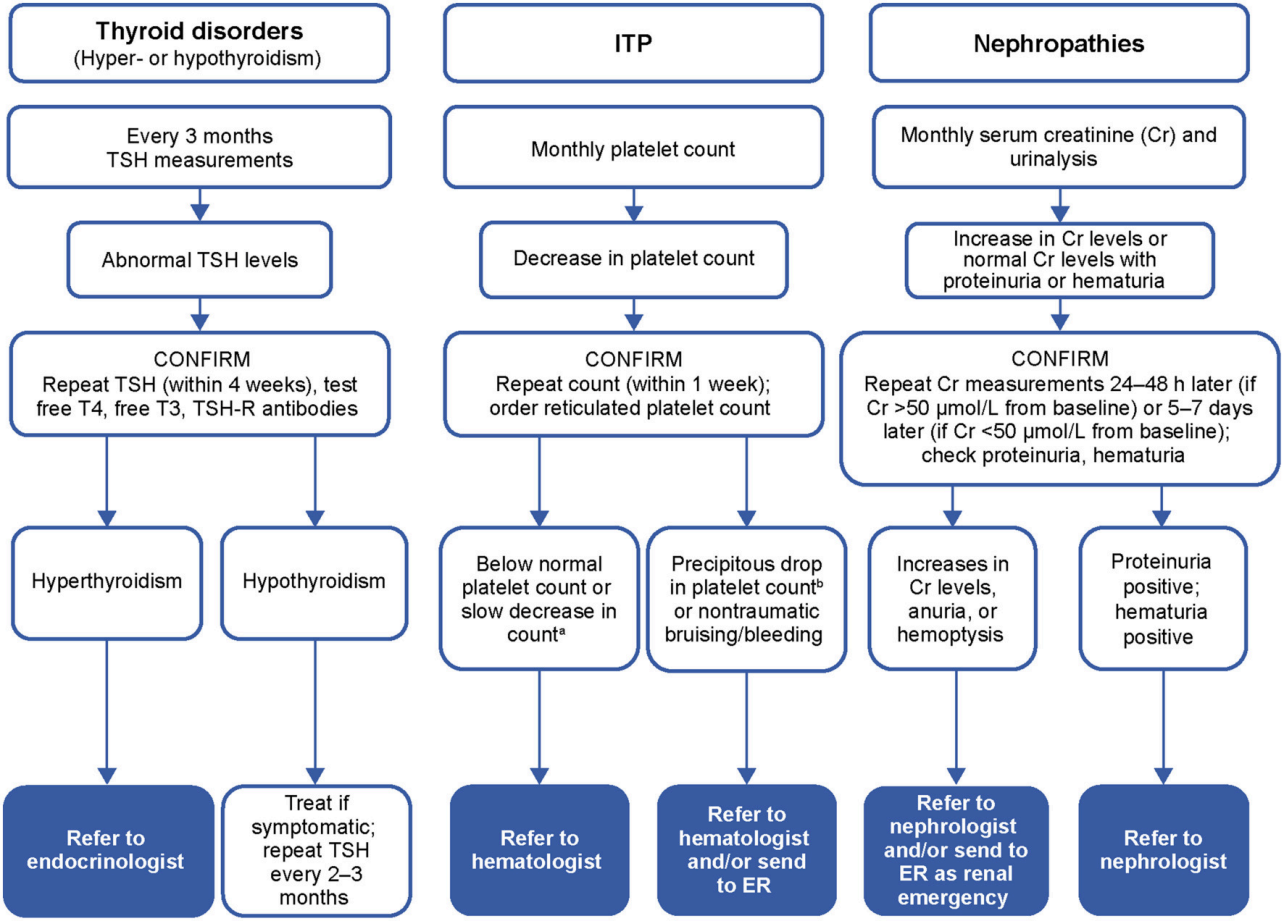
Overview of the laboratory testing plan for autoimmune disorder risk monitoring. ^a^Approximately 10% decrease in 3 successive measurements or >30% decrease from previous month or reticulated platelet count is above normal; ^b^Platelet count < 50 × 10^9^/L. T3, triiodothyronine; T4, thyroxine; TSH, thyroid-stimulating hormone; TSH-R, thyroid-stimulating hormone-receptor; ER, emergency room.

A recent task force of MS specialists, hematologists, nephrologists, and endocrinologists from Belgium issued practical recommendations on the management and daily clinical care of patients in the event of abnormal findings following treatment with alemtuzumab (Figure 4) (54–56). In patients with thyroid dysfunction associated with alemtuzumab treatment, a watch-and-wait approach is recommended if thyroid-stimulating hormone levels increase but free thyroxine (T4) levels remain normal, with testing every 6 weeks (54). In case of elevated thyroid-stimulating hormone, if free T4 is decreased, or if symptoms/signs of hypothyroidism are present, or if baseline serum thyroperoxidase antibody status is positive, treatment with synthetic T4 is indicated. In case of increased free T4 (>1.5-fold the upper limit of normal) or symptoms/signs of hyperthyroidism, symptomatic treatment with propranolol should be started.

In patients with clinical signs suggestive of bleeding after alemtuzumab, a complete blood count (CBC) must be obtained immediately (55). Any steep decrease of 50% or more from previous value but still above 100,000/μL must prompt an immediate recheck of the CBC to exclude pseudothrombocytopenia (platelet aggregates), with a referral to the hematologist if ITP is confirmed. A CBC between 30,000 and 100,000/μL and no bleeding symptoms should prompt a recheck within 48–72 h, and a hematologist should be contacted to discuss referral. Active bleeding or platelet count below 10,000/μL make treatment mandatory.

Isolated microscopic hematuria can be an initial sign of alemtuzumab-related anti-glomerular basement membrane disease (56). Weekly urinalysis is recommended to check for isolated or persistent hematuria. Isolated gross hematuria with urine discoloration must be reported immediately to the patient's neurologist or GP for follow-up blood and urine testing. Persistent hematuria must prompt a referral to a routine nephrology consultancy as soon as possible to test for anti-glomerular basement membrane antibodies. An immediate referral to a nephrologist is warranted in case of persistent hematuria with increased serum creatinine. A proteinuria finding above 0.2 grams protein per gram of creatinine needs further investigation, with referral to a nephrologist for a non-urgent consultation once urinary tract infection is excluded.

## Discussion

Effective disease management of chronic conditions such as MS requires a collaborative approach between HCPs and patients, and a multidisciplinary team of support staff. Treatment with alemtuzumab has been shown to reduce relapse rates, improve disability, and decrease MRI lesion activity and brain volume loss in patients with active RRMS over at least 7 years, with many patients receiving no additional alemtuzumab courses or other disease-modifying therapies (11–15). Improvements in disability are associated with improvements in patients' quality of life and work productivity, and decreases in healthcare costs (57, 58).

However, these benefits are associated with potential side effects. There is an increased risk for autoimmune events in patients treated with alemtuzumab (mainly thyroid events, but also ITP and nephropathies), which requires a specific monitoring regimen. These events can be managed if diagnosed early. AEs with other disease-modifying therapies have associated risks that remain constant or even increase with repeated exposure to drug (59–61). Other rare autoimmune side effects (e.g., alopecia, acquired hemophilia A, and neutropenia) have also been reported in patients treated with alemtuzumab (23, 24, 62). Implementing a comprehensive management program may improve outcomes in patients treated with alemtuzumab and maximize the therapeutic benefit as shown by the consistent safety profile in patients with active RRMS over 7 years (14, 15). Recent publications have outlined practical recommendations for managing these autoimmune events after alemtuzumab through careful analysis of monthly laboratory results (54–56, 63).

Several tools and strategies have been developed to implement the monitoring requirements and follow-up of patients treated with alemtuzumab. The use of educational materials through verbal reinforcement and support from physicians and nursing staff, via online tools, and through coordination with ancillary caregivers will increase patients' understanding of treatment benefits and risks.

Specialized nursing services that can facilitate interdisciplinary care have been beneficial, along with the use of online technologies, social media, and electronic health records and databases for management of laboratory results and patient data. These databases also enable access to patient data in real-time, which helps HCPs make informed clinical decisions.

Sustained patient outreach efforts and patient engagement by caregivers, including social workers, may improve patient adherence to monitoring, improve coping skills, and have a positive impact on patients' overall quality of life. The best practice recommendations described in this article are based on experiences in the real-world clinical settings, and may help reduce the burden of monitoring for both patients and HCPs and maximize the benefits in MS patients while minimizing the risks of treatment with alemtuzumab.

## Author Contributions

All authors had full editorial control of the manuscript and provided input and approval of all drafts, including the final, submitted version.

### Conflict of Interest Statement

KB: consulting fees, speaker honorarium, and travel assistance (Sanofi). RC: consulting/speaking fees (Biogen, EMD Serono, Novartis, Roche, Sanofi, and Teva); grants/research support (MedImmune, Novartis, Roche, and Teva). AT: consulting fees (Biogen, Novartis, Roche, Sanofi, Serono, and Teva); grant/research support (Roche and Sanofi). ADB: consulting fees/fees for non-CME services from commercial interest or their agents/grant and research support (Biogen, Mallinckrodt, Novartis, Roche-Genentech, Sanofi, and Teva Neuroscience). CLG: consulting fees (Acorda Therapeutics, Bayer, Biogen, Cephalon, EMD Serono, Novartis, Pfizer, Questcor, Sanofi, Strativa, Teva, and UCB). ABe: advisory boards and/or speaker honoraria (Almirall, Bayer, Biogen, Novartis, Sanofi, and Teva); grant support (Almirall, Associazione San Luigi Gonzaga ONLUS, Bayer, Biogen, Fondazione per la Ricerca Biomedica ONLUS, the Italian Multiple Sclerosis Society, Merck, Novartis, Sanofi, and Teva). ABo: consulting fees and/or fees for non-CME services (Biogen, Mallinckrodt, Medtronic, Novartis, Sanofi, and Teva). EGC: advisory boards and/or speaker honoraria (Biogen, Genzyme, Merck, Novartis, Roche, and Teva); unrestricted research grants (Genzyme and Novartis). JdS: consulting and/or speaking fees, advisory board, and grant/research support (Sanofi). DD: speaker honorarium and travel assistance (Sanofi) and advisory boards (Biogen Idec, Merck Serono, Novartis, Roche, and Sanofi). MH: clinical investigator and/or speaker (Actelion, Alexion Pharmaceuticals, Alvogen, Bayer, Biogen, Merck, Novartis, Pliva/Teva, Roche, and Sanofi). J-ML: advisory board and speaker bureaus (Acorda Therapeutics, Genzyme, Novartis, and Teva). VL: consulting and/or speaker honoraria (Bayer, Biogen, Merck Serono, Novartis, Roche, Sanofi, and Teva) with approval by the HR-Department, Cologne General Hospital, University of Cologne. SGM: received honoraria for lecturing and travel expenses for attending meetings (Almirall, Amicus Therapeutics Germany, Bayer HealthCare, Biogen, Celgene, Chugai Pharma, Diamed, Genzyme, MedDay Pharmaceuticals, Merck Serono, Novartis, Novo Nordisk, ONO Pharma, QuintilesIMS, Roche, Sanofi-Aventis, and Teva), and research funding (Almirall, Amicus Therapeutics Germany, Biogen, Deutsche Forschungsgesellschaft [DFG], Diamed, Else Kröner Fresenius Foundation, Fresenius Medical Care, Genzyme, German Academic Exchange Service, German Foundation Neurology, German Ministry for Education and Research [BMBF], Hertie Foundation, Interdisciplinary Center for Clinical Studies [IZKF] Muenster, Merck Serono, Novartis, ONO Pharma, Roche, and Teva). CO-G: advisory board/speaker (Biogen, Genzyme, Merck, Novartis, Roche, and Teva). PP: consulting fees and/or speaking fees for non-CME services from commercial interest or their agents (Acorda, Biogen, Mallinckrodt, and Sanofi) and consulting fees (AIMS, CMSC CME program, and IOMSN). CV: advisory board (Sanofi). TZ: consulting and/or speaking fees (Almirall, Bayer, Biogen Idec, Genzyme, Merck, Novartis, Roche, and Teva) and grant/research support (Biogen Idec, Genzyme, Novartis, and Teva). DPB: employee of Sanofi with ownership interest. BVW: advisory board and/or speaker honoraria (Biogen, Merck, Novartis, Roche, and Sanofi) and grant/research support (Biogen, Merck, Novartis, and Sanofi). The funders played no role in the study design, the collection, analysis or interpretation of data, the writing of this paper or the decision to submit it for publication.
